# Observational Evidence for Unintentional Weight Loss in All-Cause Mortality and Major Cardiovascular Events: A Systematic Review and Meta-Analysis

**DOI:** 10.1038/s41598-018-33563-z

**Published:** 2018-10-18

**Authors:** Fernanda do Carmo De Stefani, Priscila Saia Pietraroia, Miguel Morita Fernandes-Silva, José Faria-Neto, Cristina Pellegrino Baena

**Affiliations:** Pontifícia Universidade Católica do Paraná (PUCPR), Rua Imaculada Conceição, Curitiba, Brazil

## Abstract

The obesity paradox has been described in several observational cohorts and meta-analysis. However, evidence of the intentionality of weight loss in all-cause deaths and major cardiovascular events (MACE) in prospective cohorts is unclear. We analysed whether involuntary weight loss is associated with increased cardiovascular events and mortality. In a systematic review, we searched multiple electronic databases for observational studies published up to October 2016. Studies reporting risk estimates for unintentional weight loss compared with stable weight in MACE and mortality were included. Fifteen studies met the selection criteria, with a total of 178,644 participants. For unintentional weight loss, we found adjusted risk ratios (RRs) with confidence intervals (CIs) of 1.38 (95% CI: 1.23, 1.53) and 1.17 (95% CI: 0.98, 1.37) for all-cause mortality and MACE, respectively. Participants with comorbidities, overweight and obese populations, and older adults yielded RRs (95% CI) of 1.49 (1.30, 1.68), 1.11 (1.04, 1.18), and 1.81 (1.59, 2.03), respectively. Unintentional weight loss had a significant impact on all-cause mortality. We found no protective effect of being overweight or obese for unintentional weight loss and MACE.

## Introduction

The prevalence of obesity is increasing in most countries^1^, and has led to a global impact as it has been associated with all-cause mortality^2^, onset of serious diseases such as diabetes^3^ and cancer^4,5^, and increased cardiovascular risk^6^. There is continued interest in ideal weight ranges among epidemiological studies. The “U-” or “J-shaped” curves found in analyses of large cohorts indicate extremes of body mass index (BMI), underweight and obesity, associated with increased mortality in adult populations, regardless of sex or race^7–10^.

Randomized controlled trials have shown that intentional weight loss decreases mortality^11–13^; conversely, observational studies report that being overweight might be protective for some outcomes^14^. A higher BMI seems to be beneficial, especially in certain circumstances or populations, such as among older adults^15,16^ and patients with heart failure^17^, chronic obstructive pulmonary disease^18^, and chronic kidney disease^19,20^. Similarly, although the benefits of weight loss for decreasing some cardiovascular risk factors (blood pressure, glucose, and lipid levels) have already been established^21–24^, several observational studies have shown that weight reduction is associated with increased mortality from all causes and from cardiovascular disease^25–28^.

However, it has been noted that the variable “intentionality of weight loss” has not been adequately explored in most cohorts, especially with respect to methodological aspects^26,29–34^, which can possibly lead to a false positive association of weight loss and these adverse consequences. Unintentional weight loss is the involuntary decline in total body weight over time and it is mostly caused by malignant diseases, chronic organ diseases, drug-induced weight-loss or psychological disorders but up to one quarter of all cases have no identifiable cause, despite extensive investigation since its pathophysiology is poorly understood^35^.

Based on this gap in the analytical evidence regarding the intentionality of weight loss and its association with MACE and mortality, we conducted a systematic evaluation of current observational studies to estimate the increased risk of MACE or death linked with unintentional weight loss in general populations.

## Methods

### Search strategy

We performed a systematic electronic search in the Medline/PubMed, Web of Science, SciELO, and LILACS databases of studies published up to October 2016 to identify articles that studied the effect of unintentional weight loss on MACE and all-cause mortality. To perform a thorough search of the databases, the following Medical Subject Heading (MeSH) terms were used: prospective study, observational study, cohort study, follow-up study, body mass index, body weight change, stroke, myocardial infarction, acute coronary syndrome, coronary disease, incidence, and death. Full search strategies and keywords are summarized in the Supplementary Method. We used a pre-defined protocol, in accordance with the standards of quality for reporting Meta-analysis of Observational Studies in Epidemiology (MOOSE) guidelines^36^.

### Eligibility criteria

The inclusion criteria were: English language cohort studies; studies conducted with adult populations; studies that reported BMI and weight loss as numeric variables or percentages; studies that distinguished intentional from unintentional weight loss; studies that reported risk estimates for MACE (stroke, myocardial infarction, acute coronary syndrome, and cardiovascular death) or/and all-cause mortality. The intentionality of weight loss was described in the studies; unintentional weight loss was defined as any weight loss in the absence of self-reported action to try to lose weight, which may include diet, physical activity, use of medications, or by medical recommendation.

The exclusion criteria were letters, abstracts, conference proceedings, clinical trials, cluster trials, and randomized controlled trials studies; studies involving children and animals; studies conducted among bariatric surgery populations; cohorts that included only populations with poor health status at baseline (cancer, heart failure, diabetes, chronic kidney disease, or other diseases). We excluded randomized controlled trials with weight loss interventions because we assumed that all participants included in those trials intended to lose weight.

After removal of duplicates, two authors independently reviewed each title and abstract to determine whether the study met the inclusion and exclusion criteria. Disagreements about any selected items were resolved through discussions and by a third author if needed. After the initial screening, articles were chosen based on their complete text. Reference lists of the selected articles were searched manually for additional publications. The authors were contacted directly for any additional information and/or unpublished studies. If results from a single study were reported more than once, the study with the more complete set of data was included in the analysis.

### Quality assessment

To evaluate the quality of studies, we customized the Newcastle-Ottawa Scale for cohort studies^37^. For “Selection” criteria, one star was given for each of the following items: good representativeness of the exposed cohort (general population, whole population of an area or a representative sample, non-hospitalized subjects), good representativeness of the non-exposed cohort (people with stable weight within the same cohort), at least two measures of BMI during follow-up, and participants without comorbidities at baseline. For “Comparability”, one star was given for studies that adjusted for smoking (or if this population was excluded) and another star for those that used a threshold for significant weight loss of up to a 3% or 4-kilogram change^38^. For “Outcome”, one star was given for each of the following: studies with at least three years of valid follow-up, studies with no more than 10% of participant baseline information missing, and studies in which outcomes were registered in national records. A total of nine stars could be achieved for each study. Two reviewers independently assessed the quality of the manuscripts. Quality was assigned as suboptimal for studies with less than four stars (Supplementary Table S1).

### Data extraction and analysis

Two authors independently performed extraction of data from the articles through use of a data collection form, which was designed prior to the database searches. The main study and participant characteristics recorded were first author’s last name; year of publication; name of the cohort; geographic origin; population size; participants’ sex, age and comorbidities (diabetes, cardiovascular disease, cancer, hypertension); smoking status; alcohol intake; BMI; weight loss amount (in pounds, kilograms, or percentage); whether change in weight was reported or measured; time of follow-up; reference group; hazard ratio (HR) or relative risk (RR) and corresponding 95% confidence interval (CI) from the most fully adjusted multi-variable model; adjustment factors as potential confounders; and quality score.

### Statistical analysis

Study HRs or RRs were pooled and a random effects model was used to summarize the results, as a conservative approach. Heterogeneity among studies was measured using the I^2^ statistic. We focused on two possible outcomes: all-cause mortality and MACE, which included cardiovascular mortality or cardiovascular events (stroke, myocardial infarction, and acute coronary syndrome). Subgroup analysis was performed to compare studies by age, sex, initial BMI, and health status at baseline. Results are shown in the form of forest plots. To evaluate the effects of time of follow-up, initial BMI, and all-cause mortality risk, we conducted meta-regression analysis. A funnel plot evaluated publication bias (Supplementary Figure S1). All analyses were carried out using Stata version 14.0 (StataCorp LP, College Station, TX, USA).

## Results

### Study selection

**Figure 1:**
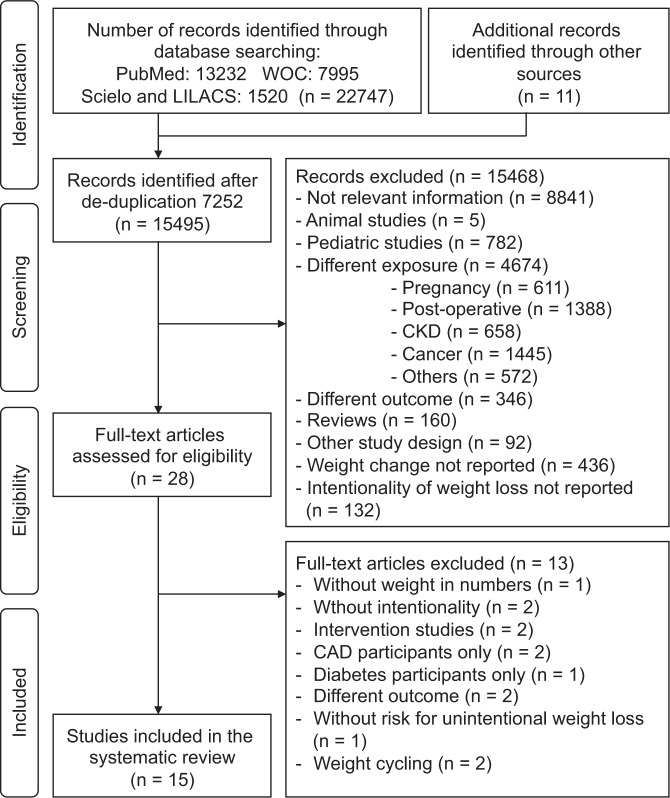
Flow Diagram of Systematic Searches in the Selection Process. Abbreviations: CAD, coronary artery disease; CKD, chronic kidney disease; WOC, Web of Science.

A total of 22 747 references were identified via electronic searches. After de-duplication and review of 11 more articles from a manual search of bibliographic ref.^28^, studies were selected for full-text reading, and 15 were included in the systematic review. Our inter-rater agreement between reviewers for inclusion criteria was k = 0,69. The reasons for excluding studies are outlined in Fig. 1.

### Study characteristics

Table 1 shows a summary of characteristics of the studies and populations eligible for inclusion in the systematic review. Nine studies were conducted in the United States^15,39–46^, one in Australia^47^, one in Finland^48^, one in Israel^25^, one in the Netherlands^49^, one in Norway^50^, and one in the United Kingdom^51^. The sample comprised 178 644 participants with reported intentionality of weight loss, 50.3% women, and mean age ranging from 42.2 to 75.3 years. Unintentional weight loss was reported in 24 995 participants. Follow-up time varied from two to 20 years. Five studies exclusively assessed men^25,39,41,43,51^, two exclusively assessed women^40,45^, and the remainder included individuals of both sexes. Regarding health status, most cohorts included some participants with comorbidities (diabetes, cardiovascular disease, cancer and hypertension), but four articles reported a complete absence of these diseases at baseline^39,40,46,48^.

**Table 1:** Characteristics of Included Studies.

First Author	Publ Year	Period	Study name	Outcome	*n Study pop*	Age (y) mean	Sex Female %	BASELINE				Curent Smoking %	*Weight Ascertainment (measured or reported)*	Time of follow up (y)	Initial BMI	*Weight Loss Amount*	Adjusted factors
								DM%	HBP%	Cancer%	CVD%						
Williamson, DF	1995	1959–1972	Cancer Prevention Study I	All-cause mortality	43,457	52	100	0	0	0	0	0	reported	12	30.6	≥9.1 kg	Age, Sex, Race, Initial BMI, Smoking, Alcohol, Cancer, Physical Activity, DM, Education, HBP, CVD
				MACE	28,388	52	100	0	0	0	0	0	reported	12	30.6	≥9.1 kg	
Wallace, JI	1995	1986–1989	Seatle VA Medical Center	All-cause mortality	247	72.9	0	19.4	50.8		27.6	21.7	measured	2	27.1	≥4%	Age, Sex
Yaari, S	1998	1963–1968	Israeli Ischemic Heart Disease Study	All-cause mortality	9,228	49.2	0	8.4	12.4	0.7	5.1	51.6	measured	18	25.5	≥5 kg	Age, Sex, Initial BMI, Smoking, Cancer, DM, HBP, High Total Colesterol
Diehr, P	1998	1989–1994	Cardiovascular Health Study	All-cause mortality	2,410	73	100			0		0	reported/measured	5	26.6	≥4.5 Kg	Age, Sex, Smoking
				All-cause mortality	1,907	73	0			0		0	reported/measured	5	26.5	≥4.5 Kg	
Williamson, DF	1999	1959–1972	Cancer Prevention Study I	All-cause mortality	49,337	51	0	0	0	0	0		reported	12	29.3	≥9.1 kg	Age, Sex, Race, Initial BMI, Smoking, Alcohol, Cancer, Physical Activity, DM, Education, HBP, CVD
				MACE	36,280	51	0	0	0	0	0		reported	12	29.3	≥9.1 kg	
French, SA	1999	1986–1992	Iowa Women’s Health Study	All-cause mortality	25,897	68	100					9.2	reported	3	27.1	≥9.1 kg	Age, Sex, Smoking, Cancer, DM, Education, HBP, CVD
				MACE	25,897	68	100					9.2	reported	3	27.1	≥9.1 kg	
Gregg, EW	2003	1989–1997	National Health Interview Survey	All-cause mortality	6,391	54.2	44.9	6.1		0.8	4.5	22.1	reported	9	29.4	Any	Age, Sex, Race, Initial BMI, Smoking, Cancer, DM, Education, CVD
Sorensen, TIA	2005	1975–1982	Finnish Twin Cohort	All-cause mortality	2,957	42.2	34.2	0		0	0		reported	18	26.6	≥1 IMC	Age, Sex, Initial BMI, Smoking, Alcohol, Physical Activity, HBP
Wannamethee, SG	2005	1978–1996	British Regional Heart Study	All-cause mortality	4,786	66.7	0	5.8	26.9	5	17.4	15	reported/measured	7	27.2	Any	Age, Sex, Smoking, Alcohol, Cancer, Physical Activity, DM, HBP, CVD
				MACE	4,786	66.7	0	5.8	26.9	5	17.4	15	reported/measured	7	27.2	Any	
Locher, JL	2007	1999–2001	UAB Study of Aging	All-cause mortality	983	75.3	49.5					13.1	reported/measured	3		≥4.5 kg	Age, Sex, Race, Smoking
Wilsgaard, T	2009	1979–1995	TromsØ Study	All-cause mortality	5,051	49.2	100			0	0	47.1	measured	11	22.5	≥2 IMC	Age, Sex, Race, Smoking, Cancer, Physical Activity, CVD
				All-cause mortality	4,881	50.8	0			0	0	47.3	measured	11	24.2	≥2 IMC	
Atlantis, E	2010	1994–2006	Melbourne Longitudinal Studies on Health Ageing	All-cause mortality	1,000		53	28.1	83.6	32.1	79.1	9	reported/measured	12		>5Kg	Age, Sex, Smoking, CVD
Lee, CG	2011	2000–2002	Osteoporotic Fractures in Men Study	All-cause mortality	4,331	72.8	0	15.7		27.6	11.7	2.9	measured	9	27.3	≥5%	Age, Sex, Race, Initial BMI, Smoking, Alcohol, Physical Activity, DM, Education
Stevens, J	2013	1987–1989	Atherosclerosis Risk in Communities Study	MACE	13,136	54	57				0	26	reported/measured	20	27.6	≥3%	Age, Sex, Race, Smoking, Alcohol, Physical Activity, Education
Wijnhoven, HAH	2014	1992–2006	Longitudinal Aging Study Amsterdam	All-cause mortality	2,645	70.4	51.1					19	reported/measured	3	26.8	Any	Age, Sex, Education

Abbreviations: CVD, cardiovascular disease; DM, diabetes mellitus; HBP, high blood pressure; MACE, major cardiovascular events.

Fourteen studies reported all-cause mortality outcomes^15,25,39–45,47–51^ and five studies reported MACE outcomes^39,40,45,46,51^. Five studies excluded participants who died within the first two years of follow-up^25,39,40,44,46^. Most studies assessed weight change retrospectively^15,25,39,40,42–47,49,50^; a few studies prospectively assessed weight change^41,48,51^ and some measured weight more than once rather than using self-reported measures^25,41,43,46^. There were no disagreement between reviewers in quality assessment scale and two studies did not attain a minimum of four points and were excluded from the meta-analysis^41,47^.

Four studies analysed 10 307 participants over 65 years old, which we considered in subgroup analysis of older adults^15,42,43,51^, with a total 3429 participants who had unintentional weight loss. Six studies^39,40,44,45,48,51^ analysed 113 503 participants with BMI ≥ 25 kg/m^2^ (obese/overweight subgroup); of these, 7757 participants had unintentional weight loss.

Baseline BMI and amount of weight loss differed among studies, and we compared the largest weight loss group with the reference group. One study used BMI gain of 1 kg/m^2^ with intention to lose weight as the reference set (RR 1.0)^50^. All the others used “weight stable, with no intention to lose weight” as the reference category for comparison, although some levels of weight change considered non-significant by study authors could be found among some “weight stable” populations.

### Unintentional weight loss and MACE

**Figure 2:**
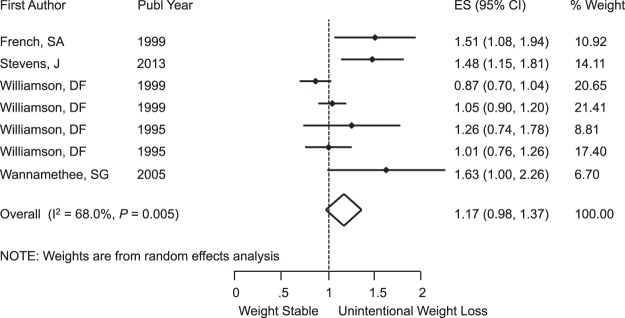
Meta-Analysis of the Effects of Unintentional Weight Loss on MACE. Abbreviations: CI, confidence interval; ES, effect size.

Unintentional weight loss was not associated with increased risk of MACE, with RR = 1.17 (95% CI: 0.98, 1.37); I^2^ = 68%; *P* = 0.005 (Fig. 2). There was heterogeneity across these studies. Subgroup analysis by sex, presence of comorbidities, and overweight/obese population showed no risk (see the Supplementary Figs S2–S4). The small number of studies was insufficient to investigate associations in the older adult subgroup with MACE.

### Unintentional weight loss and all-cause mortality

**Figure 3:**
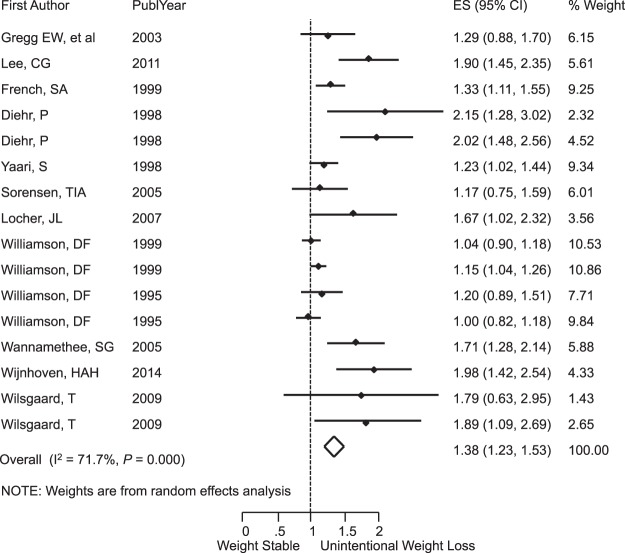
Meta-Analysis of the Effects of Unintentional Weight Loss on All-Cause Mortality. Abbreviations: CI, confidence interval; ES, effect size.

**Figure 4:**
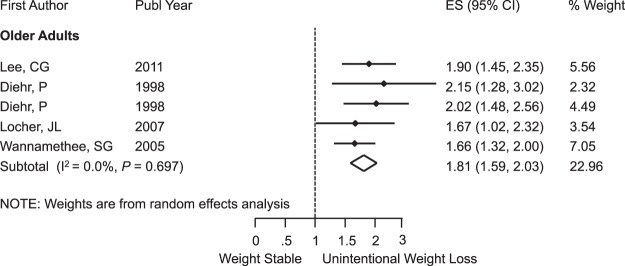
Subgroup Analysis of Older Adults With Unintentional Weight Loss and All-Cause Mortality. Abbreviations: CI, confidence interval; ES, effect size.

**Figure 5:**
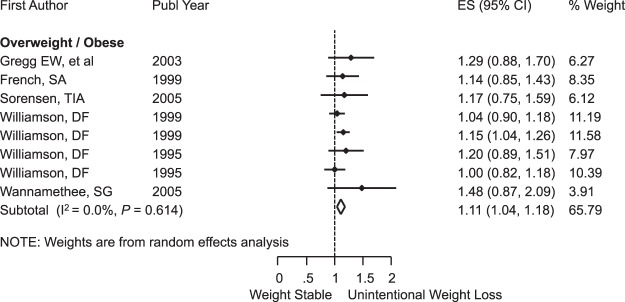
Subgroup Analysis of Obese and Overweight With Unintentional Weight Loss and All-Cause Mortality. Abbreviations: CI, confidence interval; ES, effect size.

**Figure 6:**
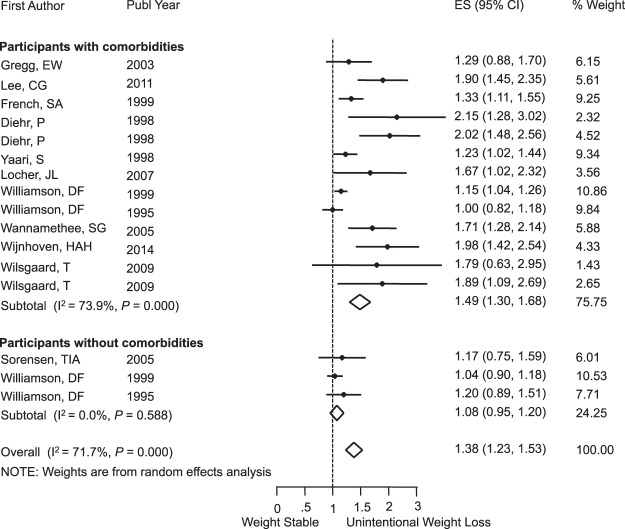
Subgroup Analysis of Participants With and Without Comorbidities for Unintentional Weight Loss and All-Cause Mortality. Abbreviations: CI, confidence interval; ES, effect size.

Unintentional weight loss was associated with significant risk of death from any cause, RR = 1.38 (95% CI: 1.23, 1.53); I^2^ = 71.7%; *P* < 0.001 (Fig. 3), and this risk was even greater among older participants with RR = 1.81 (95% CI: 1.59, 2.03); I^2^ = 0.0%; *P* = 0.69 (Fig. 4). Subgroup analysis by sex is shown in the Supplementary Fig. S5. When we looked only at overweight and obese populations at baseline, we also found the presence of mortality risk with RR = 1.11 (95% CI: 1.04, 1.18); I^2^ = 0.0%; *P* = 0.61 (Fig. 5). For participants with no comorbidities, there was no association between unintentional weight loss and mortality, RR = 1.08 (95% CI: 0.95, 1.20); I^2^ = 0.0%; *P* = 0.58 (Fig. 6).

**Figure 7:**
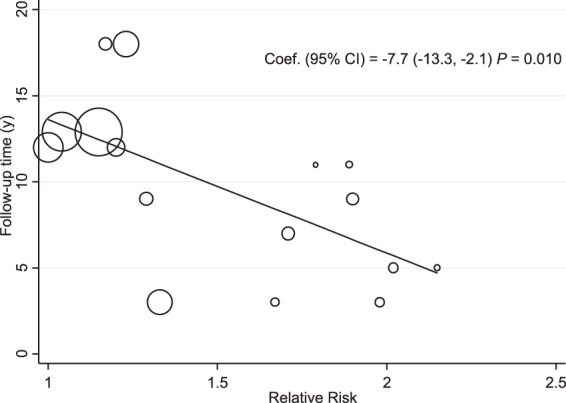
Meta-Regression Follow-up Time and All-Cause Mortality Risk. Abbreviations: CI, confidence interval; y, years.

**Figure 8:**
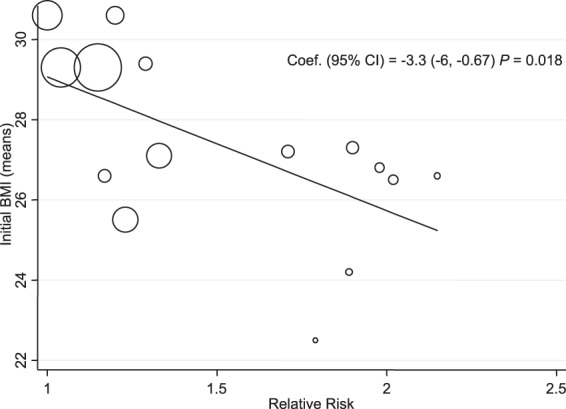
Meta-Regression of Initial BMI and All-Cause Mortality Risk. Abbreviation: BMI, body mass index; CI, confidence interval.

Meta-regression for time of follow-up showed that the longer the follow-up, the lower the risk of death due to unintentional weight loss; these data were statistically significant (*P* = 0.001) (Fig. 7). Meta-regression for initial BMI showed that the greater the initial BMI, the lower the risk of death if unintentional weight loss occurred (*P* = 0.018) (Fig. 8).

Dose–response meta-analysis was not feasible because studies reported weight loss differently, as percentage, change in BMI, or change in weight. Even after contacting the authors, no variations in participants height used to calculate BMI were reported, nor were estimates of the different categories of baseline BMI, weight loss amount or reference groups.

## Discussion

This systematic review and meta-analysis showed that unintentional weight loss was significantly associated with mortality risk in observational studies, also in overweight/obese population and especially in older adults. No protective association of unintentional weight loss was found for MACE in any of the groups studied.

Commonly, unintentional weight loss in observational studies is concomitant with smoking, which is an important risk factor for cardiovascular diseases^52^; however, confusion bias may be present in such studies^53^. Most studies controlled for smoking status to limit confounders, and this could lower the risk association between unintentional weight loss and both outcomes. The general association of unintentional weight loss with MACE remains unclear. Most previous studies that demonstrated this association were among older populations and within the context of frailty and disability^54–57^; we had insufficient data of older adults with unintentional weight loss and MACE outcomes in our review. We can suppose that people with unintentional weight loss died of non-cardiovascular causes, even in the subgroups of overweight and obese. One should use caution in interpreting the association of unintentional weight loss with MACE because of competing risk events, such as cancer mortality. Studies with MACE outcomes that used Cox proportional hazards analysis could bias the estimated rate ratios. This means that in long-term studies with seven to 20 years of follow-up, MACE could be masked by non-cardiovascular deaths that occurred before this outcome of interest^58,59^.

The association of unintentional weight loss and all-cause mortality has been published in several other studies most of them involving older subjects and populations with chronic diseases like cancer, respiratory diseases, renal dysfunction and heart failure^60–64^. However, evidence exploring the association of unintentional weight loss and all-cause mortality in obese population is lacking.

Concerning methodology, it has not been well established whether the effects of weight change depend on initial BMI^65–67^, final BMI (i.e., BMI after weight reduction)^68^, or on the magnitude of weight loss or gain^69^. Misclassification of weight loss category can occur if one adjusts for initial or final BMI^70^. Furthermore, according to recent research, if maximum BMI had been established in a weight history taken by recall rather than using baseline BMI to calculate weight loss, we may have found more obvious results in the overweight and obese subgroup, because participants in this population who had unintentional weight loss may have been misclassified into the normal weight group^71^.

It was also difficult to establish the actual short- and long-term effects of weight loss^72,73^ because follow-up times varied, and weight loss occurred during mid-life in some studies and later in life in others. In fact, long-term analysis can also be impaired by weight cycling^74^. Survival bias may be present in some cohorts included in this review, as suggested by sensitivity analysis according to time of follow-up. It is known that in observational studies, the method used to detect weight change is not always described; weight is not always measured, sometimes only reported; and weight changes before and after the follow-up period are sometimes masked^75–78^. Questionnaires used to identify weight loss attempts are sometimes subjective and differ between cohort studies. We found that in some studies, participants were asked about their intention to lose weight before weight loss occurred and in other studies, intention was queried only after weight had changed, and changes in lifestyle, diet, physical activity, or medication use were often not quantified. Defining unintentional weight loss is difficult and in studies where questions about diet and physical activity are absent, it is mostly deduced using self-reported surveys. Gregg^44^ found that participants who intended to lose weight had 24% lower mortality, regardless of whether they lost weight. That author generated the hypothesis that healthy life habits are protective, even when they do not result in reduced BMI. In addition, large intentional weight loss could mask concomitant unintentional losses in some conditions like diabetes. According to some authors, the best study design for evaluating the real impact of intentionality of weight loss is a randomized controlled trial^11,12^, especially because interventions to lose weight are not specified in observational studies and do not allow causal inference for estimating effects^79^. However, long-term clinical trials that access mortality and cardiovascular events, with large samples and groups adhering to prescribed dietary or exercise regimens, have time-bound challenges, and such trials are scarce. In addition, recruitment for intervention and control groups already implies intentional weight loss, leaving open the possibility for a lack of intentionality; this can be better analysed in observational studies.

Our review has some limitations that merit consideration. Most studies assessed weight change retrospectively, which could bring some information bias to their estimates. It is also known that measures of weight change do not distinguish between changes in lean or fat mass^80^. Declining muscle mass is associated with higher levels of cytokines and inflammatory markers^81^, and such endogenous inflammation also predicts a higher risk of mortality^82^. Considering other measures of adiposity in addition to BMI may help in the assessment of body compositional disorders^83^. The different amounts of body fat between men and women can also impair the combined analysis of these groups. Our inability to categorize different weight loss amounts for comparison may also weaken the overall results, owing to a lack of dose–response analysis, because there is no consensus on how much weight loss is clinically relevant in cohorts.

The strengths of our study include our very strict criteria for distinguishing healthy and unhealthy cohorts. A previous meta-analysis by Harrington^84^ accessed risk estimates of unintentional weight loss and all-cause mortality among both healthy and unhealthy populations up to 2008. They found higher overall mortality (RR = 1.27, 95% CI: 1.09, 1.47) for unintentional weight loss in the healthy group and no difference in mortality (RR = 1.16, 95% CI: 0.97, 1.38) in the unhealthy group, which is paradoxical. We chose to classify healthy cohorts as only when comorbidities were excluded; we also optimized a quality scale in order to exclude studies with possible information bias. It is known that participants who enter cohort studies with an underlying disease may have higher early mortality^85,86^. Although improvements in hypertension, dyslipidemia, and diabetes can be mediators by which weight loss affects vascular mortality^87–89^, we tried to clear our results of these possible confounders by providing stratified analysis of subgroups without comorbidities and by extracting HRs from the most fully adjusted models with possible intermediaries in the causal pathway. It could be hypothesized that our study results in over-adjusted analysis once we used RRs from the most fully adjusted models. However, we believe that the absence of risk for unintentional weight loss and overall mortality that we have showed in a healthy population, which is also controlled for smoking, yields more reliable data. We can speculate that the 11%, 49% and 81% higher risk-association of unintentional weight loss and all-cause mortality found in overweight and obese participants, those with comorbidities, and older adults, respectively, are not only a marker of higher morbidity but provide strong evidence for clinicians to take into consideration.

In conclusion, we found that participants with unintentional weight loss had no protective risk for MACE and significant increased risk for overall mortality. A lack of information on the intentionality of weight loss could explain part of the disagreements found among studies of weight loss, obesity, and mortality. Careful attention should be given to individuals with suspected unintended weight loss, particularly in overweight and obese, older adult, or unhealthy populations. Observational studies on weight loss with mortality or cardiovascular event outcomes should consider the intentionality factor in order to avoid important bias in the weight effect estimates of these major clinical events.

## Electronic supplementary material

Supplementary Material

## Data Availability

All data generated or analysed during this study are included in this published article (and its Supplementary Information files).
