# Supplementary material for: Observational Evidence for Unintentional Weight Loss in All-Cause Mortality and Major Cardiovascular Events: A Systematic Review and Meta-Analysis
Source: Sci Rep. 2018 Oct 18;8:15447. doi: 10.1038/s41598-018-33563-z (PMC6194006; doi:10.1038/s41598-018-33563-z)
Supplement: Supplementary file 1 — Supplementary Material [file 41598_2018_33563_MOESM1_ESM.pdf]

Observational Evidence for Unintentional Weight Loss in All-Cause Mortality and Major Cardiovascular Events: A Systematic Review and Meta-Analysis

Fernanda do Carmo De Stefani*[1, 2], Priscila Saia Pietraroia[1], Miguel Morita Fernandes-Silva[1], José Faria-Neto[1], Cristina Pellegrino Baena[1]

1 Pontifícia Universidade Católica do Paraná (PUCPR), Rua Imaculada Conceição, Curitiba, Brazil.

2 Corresponding Author: fe_destefani@yahoo.com / +55 (41) 999821324

SUPPLEMENTARY MATERIAL

Supplementary Method – Search Strategies

1. Pubmed/ Medline – 13232 articles

((((((((Prospective Stud*[MeSH Terms] OR Prospective Stud*[tiab] OR observational Stud*[MeSH Terms] OR observational Stud*[tiab] OR cohort Stud*[MeSH Terms] OR Cohort Stud*[tiab] OR Follow-up Stud*[MeSH Terms])))) AND ((body mass index[tiab] OR body mass index[tw] OR body weight change*[tiab] OR body weight change*[tw])))) AND (((death[tiab] OR stroke[MeSH Terms] OR stroke[tiab] OR myocardial infarction[MeSH Terms] OR myocardial infarction[tiab] OR acute coronary syndrome[MeSH Terms] OR acute coronary syndrome[tiab] OR coronary disease[MeSH Terms] OR coronary disease[tiab] OR incidence[MeSH Terms] OR incidence[tiab]))))))

2. Web of Science – 7995 articles

("Prospective Stud*" OR "observational Stud*" OR "cohort Stud*") AND ("body mass index" OR "body weight change*") AND ("death" OR "stroke" OR "myocardial infarction" OR "acute coronary syndrome" OR "coronary disease" OR "incidence")

3. BVS/Lilacs/Scielo – 1520 articles

"Prospective Study" OR "observational Study" OR "cohort Study" AND "body mass index" OR "body weight change" AND "death" OR "stroke" OR "myocardial infarction" OR "acute coronary syndrome" OR "coronary disease" OR "incidence"

FIRST AUTHOR	SELECTION (max 1*)				COMPARABILITY (max 2*)	OUTCOME (max 1*)			TOTAL STARS
	Selection of the exposed cohort	Selection of the non exposed cohort	Ascertainment of exposure	Participants without comorbidities at baseline	Comparability of cohorts on the basis of the design or analysis	Follow-up time	Adequacy of follow-up	Assessment of outcome	
Gregg, EW	*	*			*	*	*	*	**6**
Lee, CG			*		**	*		*	**5**
French, SA	*	*			**			*	**5**
Diehr, P	*	*			**	*	*		**6**
Yaari, S	*	*	*		**	*	*	*	**8**
Stevens, J	*	*	*	*	**	*		*	**8**
Sorensen, TIA	*	*		*	*	*	*	*	**7**
Locher, JL					**		*	*	**4**
Williamson, DF	*	*		*	**	*		*	**7**
Williamson, DF	*	*		*	**	*		*	**7**
Wallace, JI			*		*				**2**
Wannamethee, SG	*	*			*	*		*	**5**
Wijnhoven, HAH	*	*					*	*	**4**
Wilsgaard, T	*	*	*	*	*	*		*	**7**
Atlantis, E					**	*			**3**

Supplementary Table S1**.** Quality of Included Studies by Newcastle-Ottawa Scale

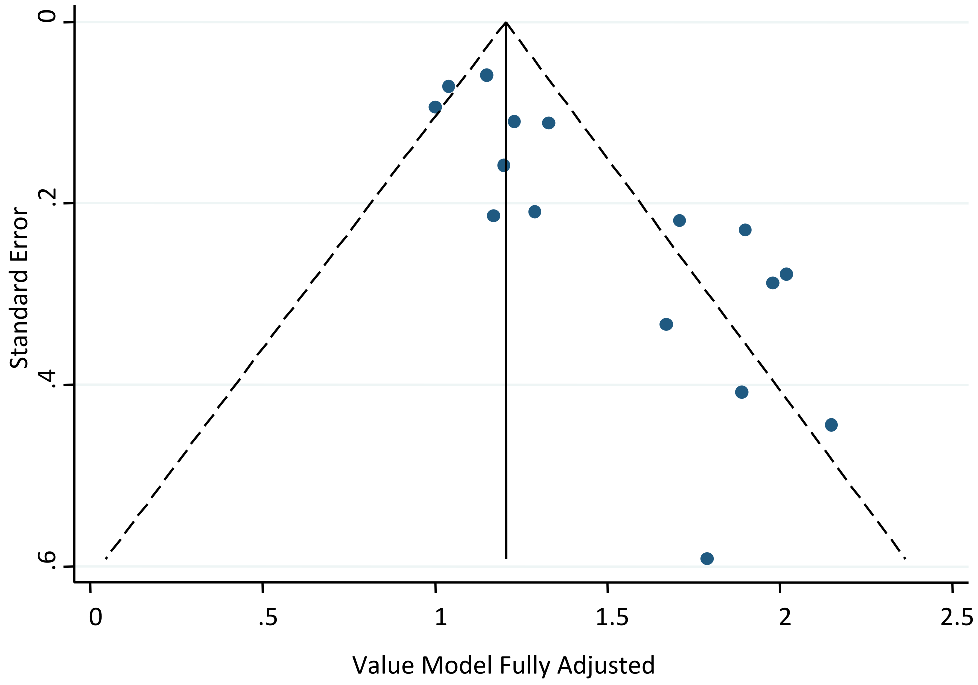

Supplementary Figure S1. Funnel Plot of the studies included in the meta-analysis for all-cause mortality

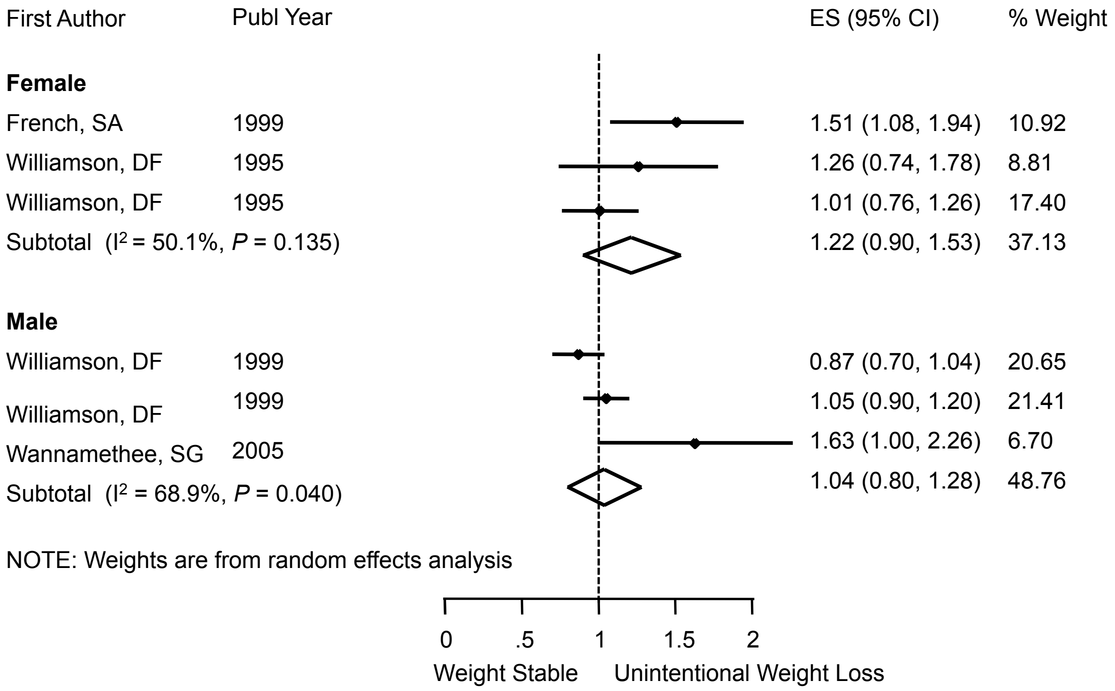

Supplementary Figure S2. Subgroup Analysis of Unintentional Weight Loss and MACE by sex

Abbreviations: CI, confidence interval; ES, effect size.

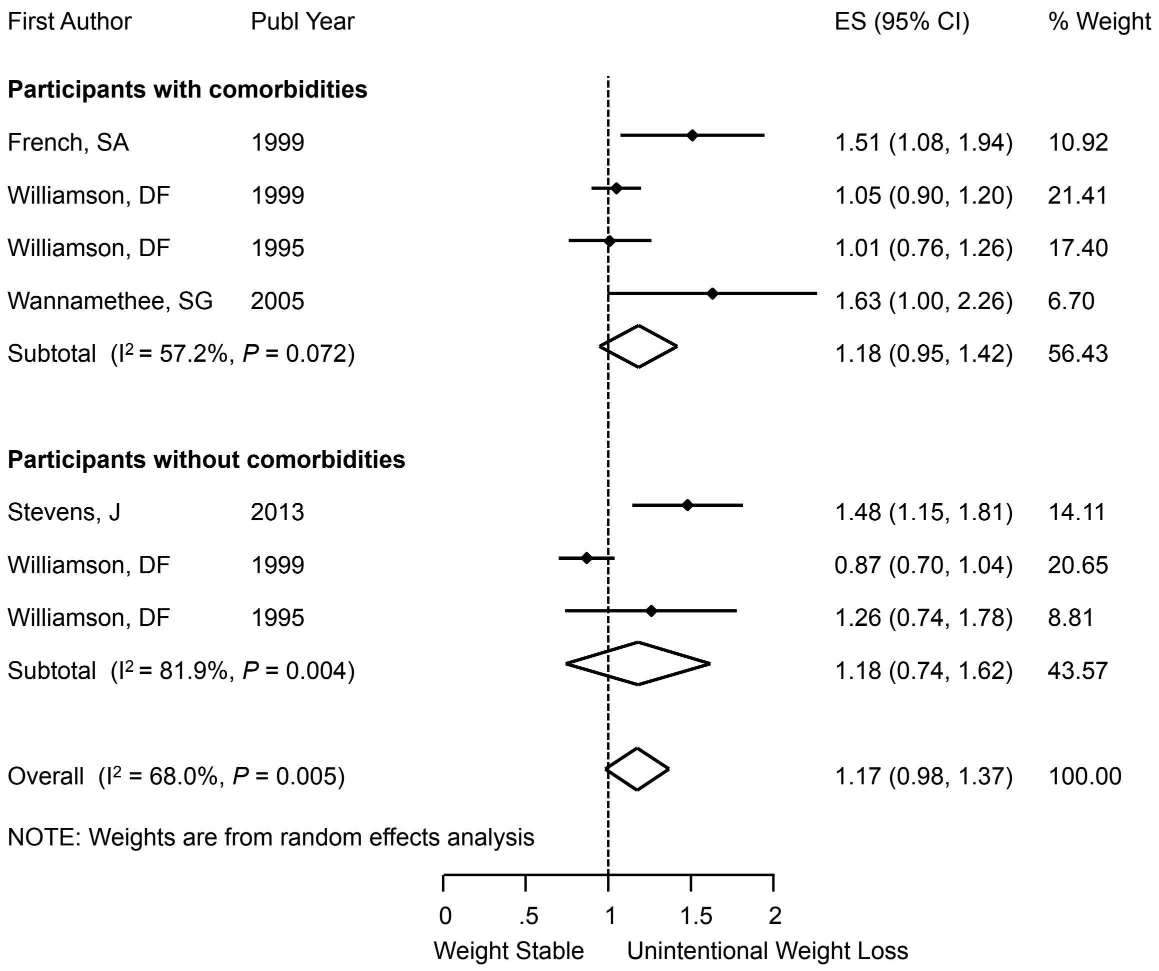

Supplementary Figure S3. Subgroup Analysis of Participants With and Without Comorbidities With Unintentional Weight Loss and MACE

Abbreviations: CI, confidence interval; ES, effect size.

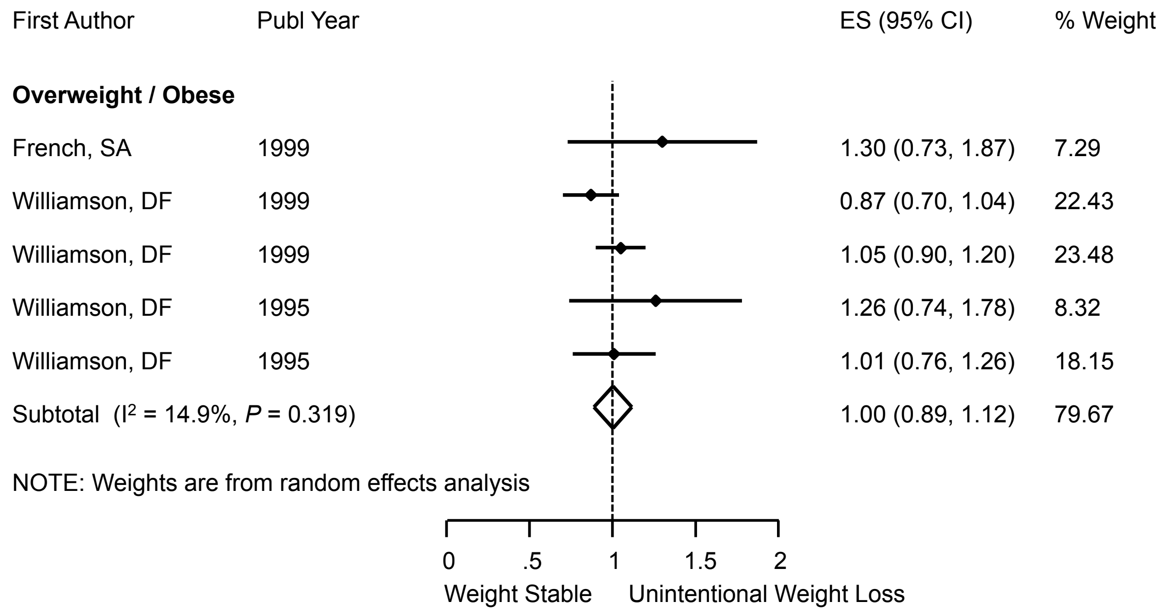

Supplementary Figure S4. Subgroup Analysis of Overweight and Obese Population With Unintentional Weight Loss and MACE

Abbreviations: CI, confidence interval; ES, effect size.

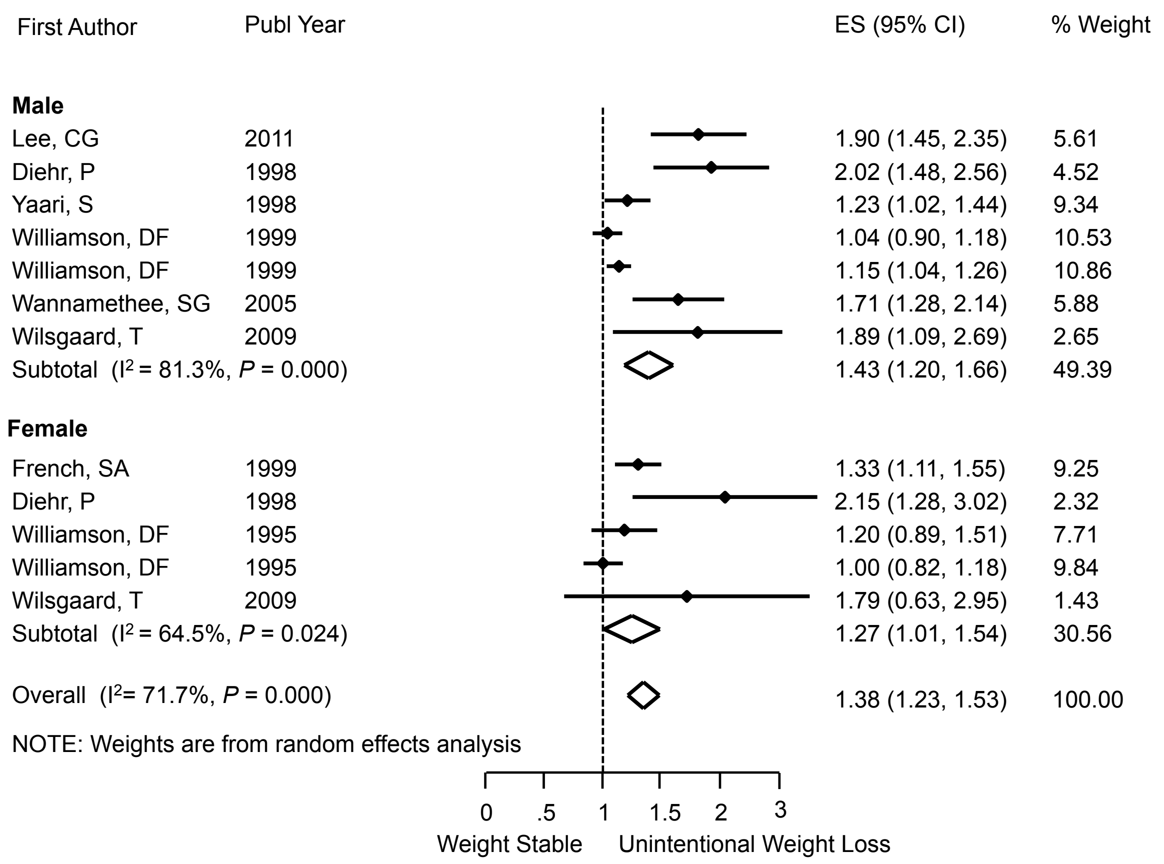

Supplementary Figure S5. Subgroup Analysis of Unintentional Weight Loss and All-Cause Mortality by sex

Abbreviations: CI, confidence interval; ES, effect size.
